# Spatial analysis and prediction of psittacosis in Zhejiang Province, China, 2019–2024

**DOI:** 10.3389/fpubh.2025.1604018

**Published:** 2025-07-02

**Authors:** Zheyuan Ding, Haocheng Wu, Chen Wu, Kui Liu, Qinbao Lu, Xinyi Wang, Tianying Fu, Junjie Li, Ke Yang, Queping Song, Junfen Lin

**Affiliations:** ^1^Zhejiang Provincial Center for Disease Control and Prevention, Hangzhou, Zhejiang, China; ^2^Hangzhou Medical College, Hangzhou, Zhejiang, China

**Keywords:** psittacosis, epidemiological characteristics, spatial analysis, Kriging interpolation, prediction map

## Abstract

**Background:**

The emergence of advanced diagnostic techniques and improved disease surveillance have led to increased recognition of psittacosis cases in recent years. This study aimed to characterize the epidemiological patterns and spatiotemporal distribution of psittacosis in Zhejiang Province, China, and to identify high-risk clusters through predictive modeling.

**Methods:**

We conducted a comprehensive analysis of reported psittacosis cases in Zhejiang Province from January 2019 to June 2024. Demographic characteristics and seasonal trends were systematically analyzed. Spatial epidemiological methods, including spatiotemporal distribution mapping, spatial autocorrelation analysis, and Kriging interpolation, were employed to identify disease hotspots and predict risk areas.

**Results:**

During the study period, 315 psittacosis cases were reported, with an annual average incidence rate of 0.0914 per 100,000 population, showing a significant increasing trend. The geographic distribution of cases expanded over time. More cases were reported in winter. Cases demonstrated a male predominance (sex ratio 1.1:1) with a median age of 64 years. Occupational analysis revealed farmers as the most affected group (52.4%). Spatial analysis identified significant clustering (*Moran's I* = 0.5428, *P* < 0.001), with high-incidence areas concentrated in western and central regions. Kriging interpolation predicted the highest disease risk in western Zhejiang, followed by central, southwestern and parts of northern regions. Western and southwestern regions had high risks of cluster.

**Conclusions:**

Our findings demonstrate a concerning upward trend in psittacosis incidence with expanding geographic distribution in Zhejiang Province. The identification of high-risk clusters in western, central, and northern regions provides critical evidence for targeted public health interventions, including enhanced surveillance in agricultural communities and seasonal prevention campaigns during winter months.

## Background

Psittacosis, caused by *Chlamydia psittaci* (*C.psittaci*), is a zoonotic disease primarily transmitted through exposure to infected birds or their secretions (feces, urine, etc.) (1). The pathogen demonstrates broad avian host specificity, capable of infecting all bird species (2); however, most human cases are associated with transmission from birds of the orders psittaformes and galliformes (3). Human-to-human transmission is also possible, but thought to be rare (4, 5). Clinical presentations range from asymptomatic infection to severe systemic illness with multiorgan failure (3). There is low awareness among clinicians and the public over the years because of the low incidence and prevalence of this disease. Coupled with the need for specialized testing, under-diagnosis and underreport of psittacosis is likely to occur (6, 7). This diagnostic challenge has been substantially addressed by the advent of metagenomic next-generation sequencing (mNGS), which offers superior sensitivity, specificity, and rapid turnaround for *C. psittaci* detection (8, 9). Consequently, recent years have witnessed a marked increase in both diagnosis rates and reported cases across China, and the number of researches related with psittacosis cases has been rising for the past few years (5, 10–13).

Zhejiang Province, lying along the southeast coast of China, has a subtropical monsoon climate, which provides a suitable habitat for birds. More than 400 species of birds have been recorded in Zhejiang bird checklist (14), including many species of psittaformes and galliformes, which are potentially infected with *C. psittaci*. Documented case clusters and sporadic reports in Zhejiang (15–18) suggest active zoonotic transmission. However, existing studies predominantly focus on pathogen biology and clinical aspects, with limited spatial epidemiological investigations. Thus, this study aims to characterize the epidemiological and spatiotemporal distribution patterns of psittacosis, as well as identify high-risk clusters through predictive model in Zhejiang Province.

## Methods

### Study area

Zhejiang Province (105,500 km^2^ land area; 260,000 km^2^ sea area) is situated on China's southeastern coast, forming the southern wing of the Yangtze River Delta (19). The province comprises 11 prefecture-level cities and 90 counties, with its resident population growing from 57.37 million in 2019 to 66.27 million in 2024.

### Data collection

The data of psittacosis cases in Zhejiang was acquired from the National Notifiable Infectious Disease Reporting Information System at the China Information System for Disease Control and Prevention, a national disease reporting system established by the Chinese Center for Disease Control and Prevention. All cases were laboratory-confirmed by the detection of *C. psittaci* in bronchoalveolar lavage fluid, sputum, or blood samples via culture, PCR, or mNGS, with confirmatory clinical diagnosis by physicians. The collected information included demographic characteristics, address (county-level) and date of onset.

### Statistical analysis

Characteristics of psittacosis cases were described by temporal trends, gender, age and occupation. Spatio-temporal distribution maps of incidence in each year were drawn at county-level. It should be noted that, in 2024, 6 months (January to June) of data rather than annual data was mapped. Annual average incidence from 2019 to June 2024 was calculated as total number of cases from 2019 to June 2024 divided by the sum of total population from 2019 to 2023 and half of the population in 2024.

Global and local spatial autocorrelation analyses (20) of annual average incidence were performed using *Moran's I* statistics. The range of *Moran's I* statistic is between [−1, 1]. *I* > 0 means there is a positive spatial correlation, while *I* < 0 means a negative spatial correlation, and *I* = 0 shows no spatial correlation. A greater absolute value of *I* indicates a higher spatial autocorrelation. There are four types of local spatial connection forms, namely, high value with high value nearby (H-H), low value with low value nearby (L-L), high value with low value nearby (H-L), and low value with high value nearby (L-H). We employed Kriging interpolation (20–22) to generate the prediction map. The theoretical underpinnings of this method have been extensively detailed in various books and literatures (20, 21, 23). The reported numbers of psittacosis cases in each county did not follow a normal or log-normal distribution. Consequently, Disjunctive Kriging interpolation was employed to predict the spatial distribution of psittacosis cases in Zhejiang Province for the period spanning 2019 to June 2024. Additionally, Indicator Kriging interpolation was applied to estimate clustering probabilities. In this study, a cluster was operationally defined as an incidence rate exceeding 0.6 per 100,000 population. Detailed methodologies for spatial autocorrelation analyses (Supplementary material 1) and Kriging interpolation (Supplementary material 2) are provided. ArcGIS software was used to conduct spatial analysis, perform Kriging interpolation, and generate spatiotemporal and predictive maps.

## Results

### Epidemiological characteristics

**Figure 1:**
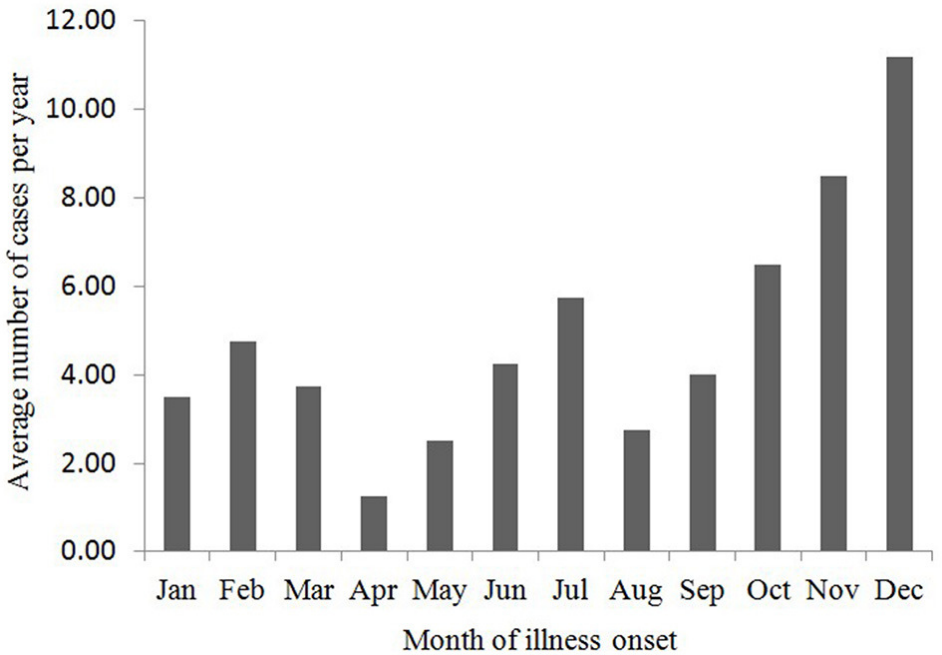
Seasonal distribution of psittacosis in Zhejiang, China, 2019 to June 2024.

By June 2024, a total of 315 cases of psittacosis had been reported in Zhejiang Province, comprising 165 males and 150 females. The earliest reported case onset was on December 10, 2019. The number of cases was relatively higher in winter, specifically in November and December (Figure 1). The age range of the cases spanned from 18 to 91 years, with a median age of 64 years. Cases aged between 50 and 79 years accounted for 83.8% of the total. Farmers represented the largest occupational group, making up 52.4% of the cases (165 out of 315; Table 1).

**Table 1:** Demographic characteristics of reported psittacosis cases in Zhejiang, China, 2019–June 2024.

	**Number of cases**	**Proportion (%)**
**Gender**		
Men	165	52.38
Women	150	47.62
**Age**		
<20	1	0.32
20~	5	1.59
30~	13	4.13
40~	22	6.98
50~	76	24.13
60~	113	35.87
70~	75	23.81
80~	10	3.17
**Occupation**		
Farmer	165	52.38
Retiree	52	16.51
Unemployed person	43	13.65
Commercial service people	22	6.98
Worker	15	4.76
Other	18	5.71

### Spatio-temporal distribution

**Figure 2:**
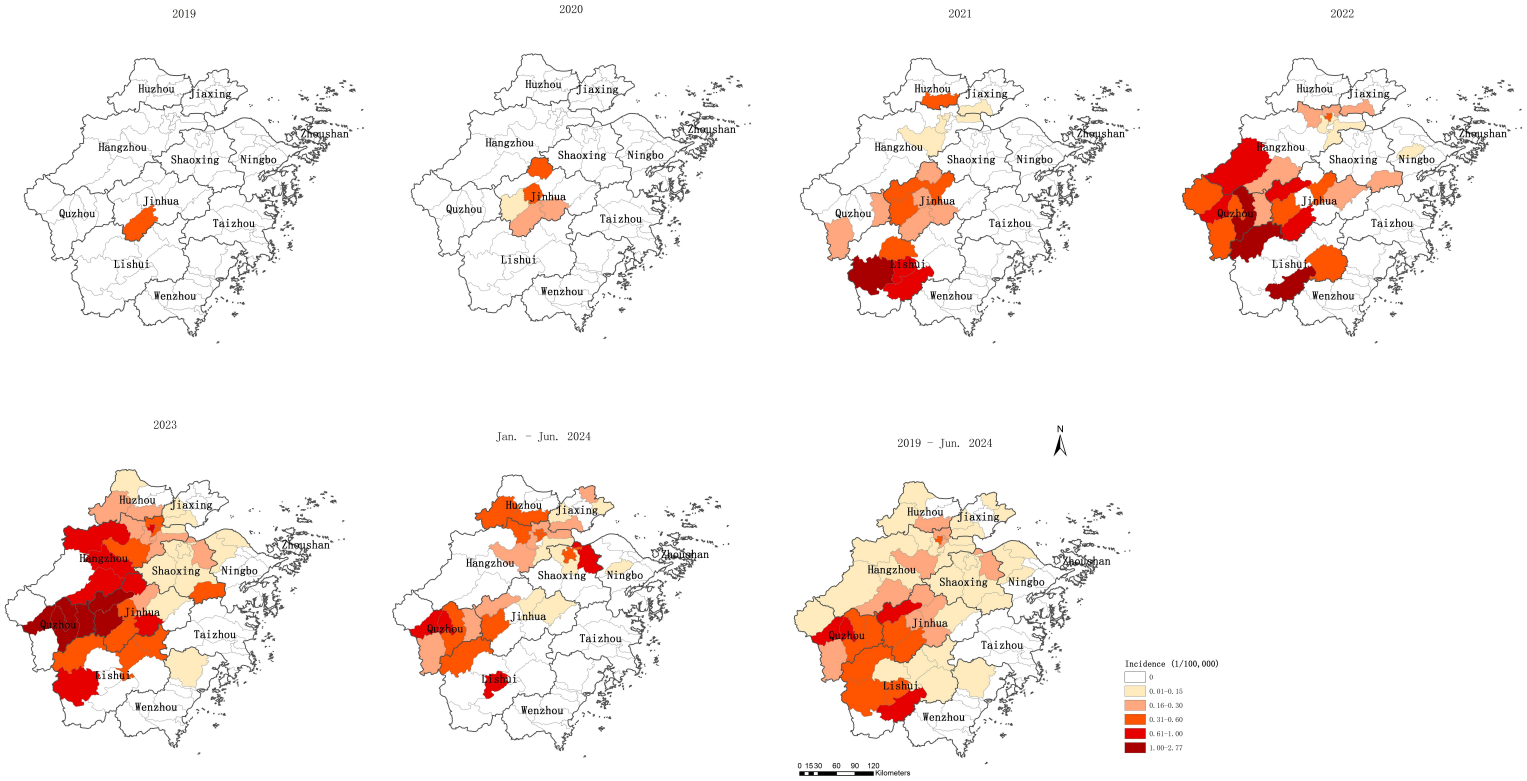
Spatio-temporal distribution of psittacosis in Zhejiang, China, 2019 to June 2024.

The incidence of psittacosis increased from 0.0035 per 100,000 in 2019 to 0.2159 per 100,000 in 2023, with an average annual incidence of 0.0914 per 100,000 over the five-and-a-half-year period. The number of counties reporting cases grew from 1 in 2019 to 5 in 2020, 19 in 2021, 25 in 2022, 42 in 2023, and 29 by June 2024. Similarly, the number of prefecture-level cities with reported cases expanded from 1 in 2019 to 1 in 2020, 6 in 2021, 7 in 2022, 9 in 2023, and 8 by June 2024, indicating a clear trend of expanding infected regions. The spread of reported cases shifted from central to southern, western, and northern Zhejiang. By June 2024, 52 out of 90 counties had reported psittacosis cases. Four counties in Quzhou, Jinhua, and Lishui had an annual average incidence exceeding 0.6 per 100,000. Counties with reported cases were primarily located in the central and western regions, as well as parts of the northern region, while counties without reported cases were mainly situated in the eastern and southern coastal areas of Zhejiang (Table 2, Figure 2).

**Table 2:** Reporting of psittacosis in China, 2019–June 2024.

	**Number of cases**	**Incidence (1/100,000)**	**Number of counties**	**Number of prefecture-level cities**
2019	2	0.0035	1	1
2020	8	0.0137	5	1
2021	35	0.0542	19	6
2022	59	0.0902	25	7
2023	142	0.2159	42	9
2024 (January–June)	69	0.1041	29	8
2019–June 2024	315	0.0914^*^	52	9

* Annual average incidence.

### Spatial autocorrelation

**Figure 3:**
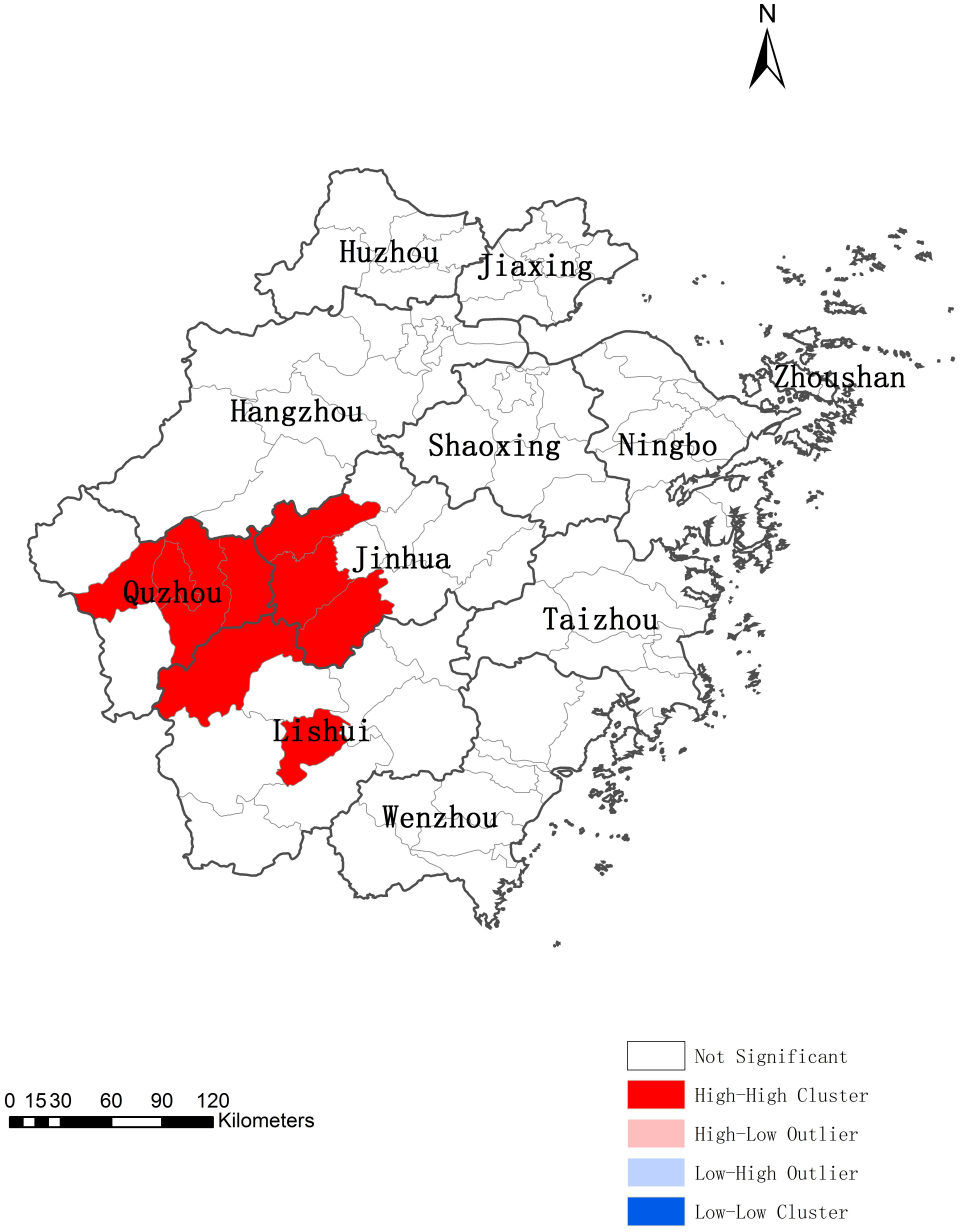
Local spatial autocorrelation of psittacosis in Zhejiang, China, 2019 to June 2024.

The spatial distribution of psittacosis cases exhibited significant global spatial positive correlation from 2019 to June 2024 (global Moran's *I* = 0.5428, *Z* = 8.1415, *P* < 0.0001). Cluster and outlier analysis identified nine counties in three prefecture-level cities (Jinhua, Quzhou, and Lishui) as high-high (H-H) clustering areas, while other counties showed no statistical significance (Figure 3). This indicates that psittacosis cases were regionally clustered in Zhejiang.

### Prediction using Kriging interpolation

**Figure 4:**
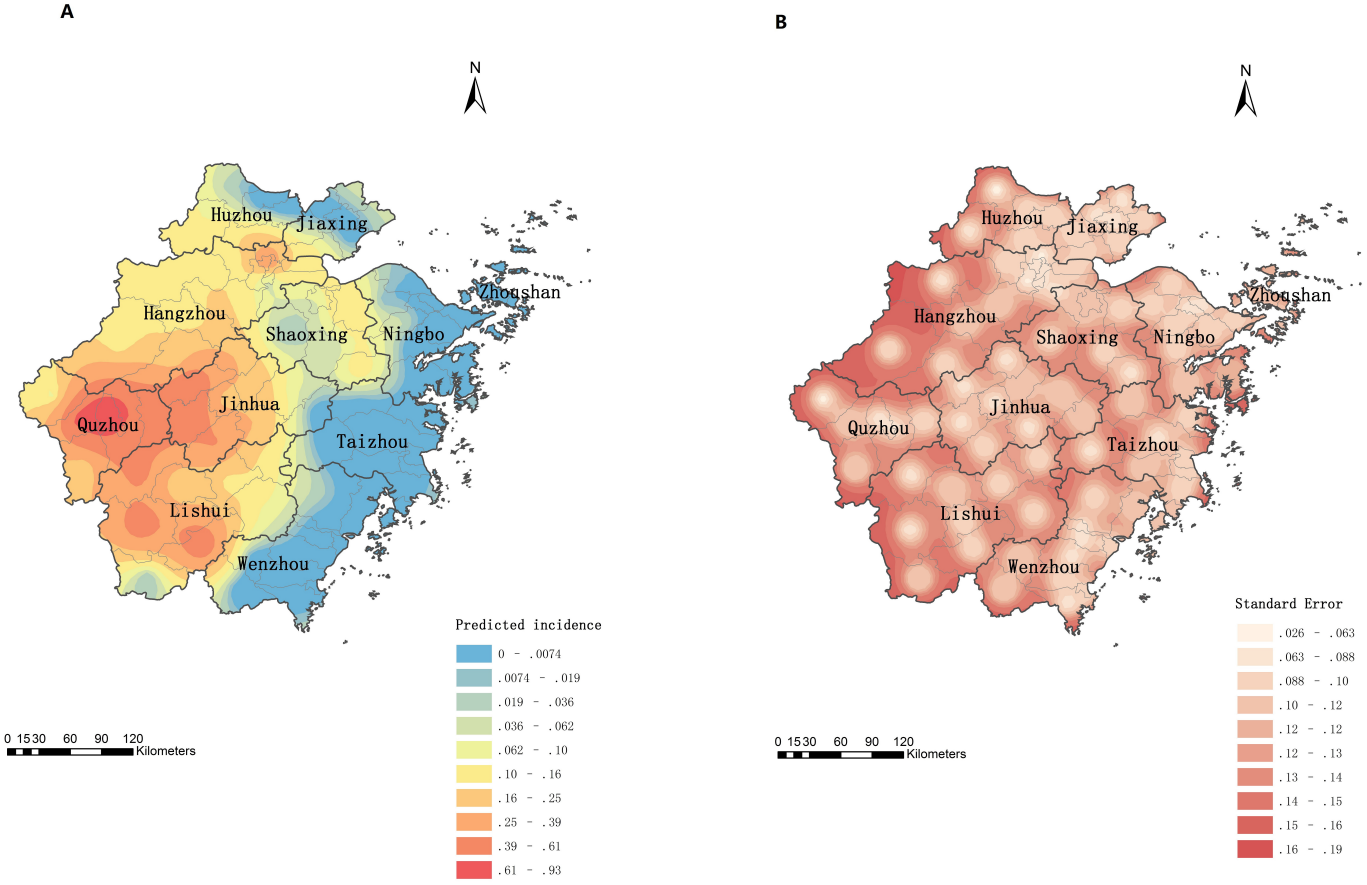
Prediction of spatial distribution of psittacosis incidence in Zhejiang, China, 2019 to June 2024. **(A)** Prediction map. **(B)** Error map.

Figure 4A presents the predicted spatial distribution of psittacosis cases in Zhejiang from 2019 to June 2024. The semivariogram model parameters were as follows: Nugget = 0, Range = 1.5036, Partial Sill = 0.8938, and Sill = 0.8938. The ratio of Partial Sill to Sill was 100%, indicating strong spatial correlation. The western region of Zhejiang, particularly Quzhou, emerged as a high-incidence area. Other regions with relatively high incidence included western Jinhua, central Lishui, and southwestern and northeastern Hangzhou. The error map (Figure 4B) revealed that marginal areas had higher prediction errors compared to central areas.

**Figure 5:**
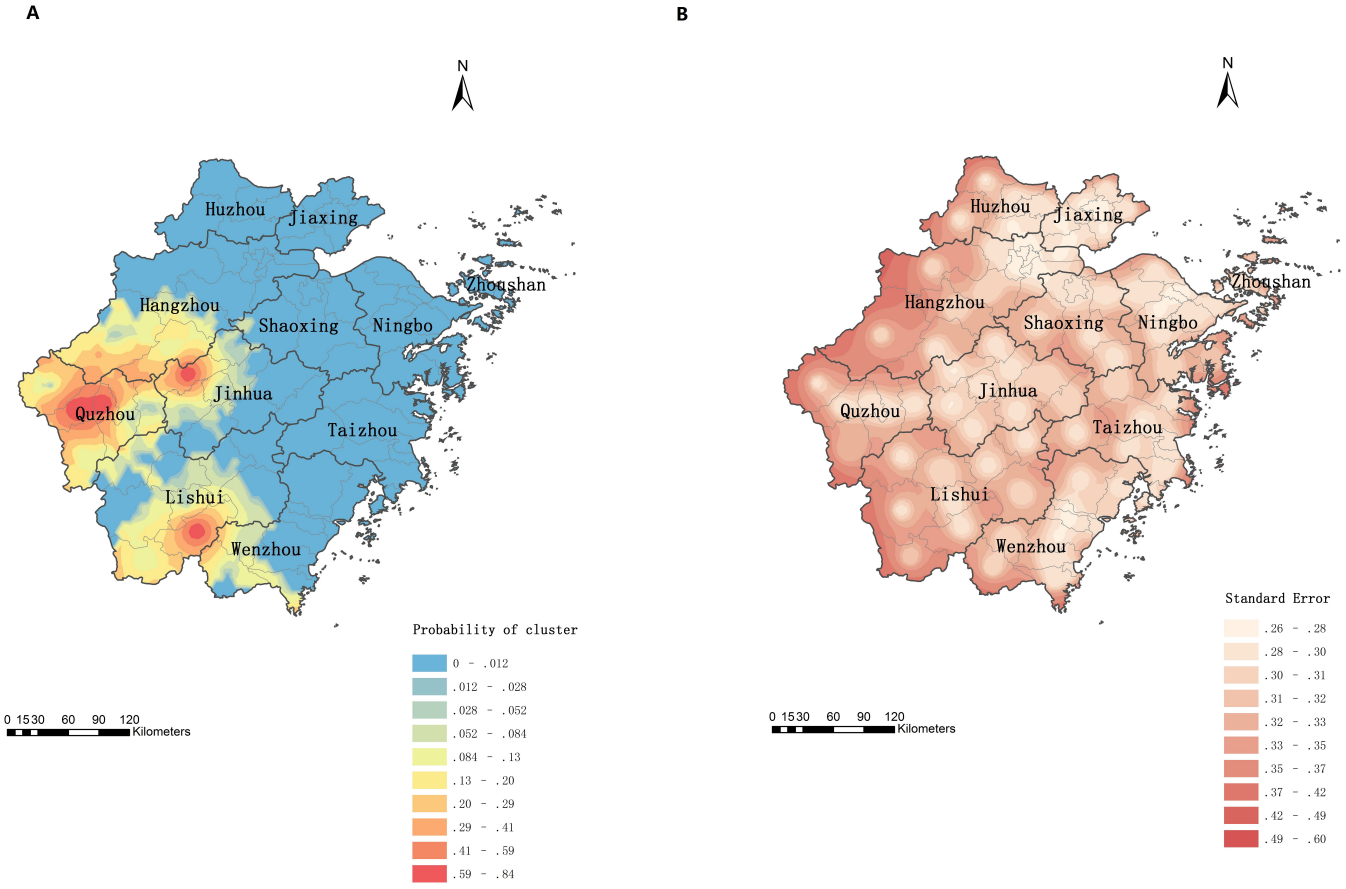
Prediction of cluster probabilities of psittacosis in Zhejiang, China, 2019 to June 2024. **(A)** Prediction map. **(B)** Error map.

Figure 5A displays the predicted probabilities of psittacosis clustering. Quzhou, northwestern Jinhua, and southern Lishui were identified as high-risk areas for psittacosis clusters. The error map (Figure 5B) showed that marginal areas of the map and county borders had higher prediction errors than other regions.

## Discussion

Our study revealed a gradual increase in the incidence of reported psittacosis cases over the past five and a half years in Zhejiang Province, with an expansion of the affected areas. Cases were predominantly distributed across most counties in the central and western regions, as well as parts of the northern region, with significant regional clustering. The predicted risks of clusters were relatively high in western and southwestern Zhejiang.

Psittacosis is generally recognized as a rare zoonotic disease worldwide. In the United States, an average of 16 cases were reported annually during 2000–2009 (24), and fewer than 100 cases per year have been reported since 2018 (7). A meta-analysis in 2017 indicated that *C. psittaci* accounted for 1.03% (95% CI 0.79–1.30) of all causative pathogens in community-acquired pneumonia (CAP) cases, with a range of 0–6.7% across studies (25). Based on these findings, it was estimated that only 4.4% (95% CI 1.6–8.2%) of symptomatic cases were notified in the Netherlands during 2012–2014 (6). These data suggest that the incidence of psittacosis is significantly underestimated globally. In China, psittacosis is not a nationally notifiable disease, and official incidence statistics are unavailable. A recent study in China showed *C. psittaci* took a proportion of 1.1% (132/11,514) of CAP cases (26), while another multicenter prospective study detected *C. psittaci* in 6.8% (15/222) of patients with severe CAP (27). These findings suggest that the incidence of psittacosis in China is also likely underestimated.

A review of 22 case reports of psittacosis in China from November 1999 to June 2021 revealed a marked increase in cases, with mNGS technology becoming the primary diagnostic method in recent years (26). Our study also showed an increasing trend in reported psittacosis cases since 2019, which may be partially attributed to the development and application of mNGS technology (28), and increased clinical awareness of psittacosis. Infection can occur throughout the year, but incidence peaks in winter, consistent with findings from other studies in China (11, 13, 26). However, this contrasts with a study in Melbourne, which reported fewer cases in winter months (29). Our findings align with previous reports that individuals aged 30–59 years are more frequently affected, children are rarely affected, and more cases are detected in men than in women (6, 29). Farmers are particularly susceptible, likely due to their frequent contact with poultry. Given the rarity of reported cases, data on seasonal, age, and gender-specific susceptibility remain limited. However, it is clear that the risk of infection is partly dependent on exposure to birds and their secretions.

The reported incidence of psittacosis varied significantly across regions, with local spatial autocorrelation evident. High-high (H-H) clusters were primarily located in three prefecture-level cities (Jinhua, Quzhou, and Lishui). Semivariogram analysis also indicated strong spatial correlation, supporting the use of Kriging interpolation. Kriging interpolation allows for the calculation of values at each point within the study area, providing a more accurate disease distribution map that is not restricted by administrative boundaries (20). The predicted incidence was higher in the western, central, southwestern, and parts of the northern regions compared to the southeastern coastal areas. The western, central, and southwestern regions are dominated by mountains and hills with lower population density, while the eastern regions are urban agglomerations with high population density and developed economies (30). Additionally, the trade of live poultry is prohibited in major cities but remains common in economically less developed areas. Therefore, residents in the west may have more opportunity to exposure to wild birds and poultry than those in the east. The high-incidence counties in the northern region are mainly urban areas of Hangzhou, the provincial capital, where medical resources are abundant (31), reducing the likelihood of misdiagnosis or underdiagnosis. Our results also exhibited that the western and southwestern regions may have higher risks of psittacosis clusters and should be prioritized for prevention and control measures by the government.

Several limitations of this study should be noted. First, the low incidence of psittacosis resulted in a small sample size. As diagnostic rates improve with new technologies, future research can accumulate more cases for more accurate analyses. Second, our study lacked detailed epidemiological information on the exposure history of psittacosis cases, preventing further analysis of infection risks associated with bird contact or human-to-human transmission. Third, the error map of Kriging interpolation indicated higher prediction errors in marginal areas. Future research incorporating data from neighboring provinces could improve prediction accuracy in these regions.

In conclusion, our study provides a comprehensive profile of the epidemiology and distribution of psittacosis in Zhejiang Province. The prediction results enhance our understanding of the disease's epidemiological characteristics and help estimate its true burden. Prevention and control measures should focus on high-incidence and high-cluster-risk areas, such as the central, western, and northern regions of Zhejiang. With the continued application of mNGS technology and increased awareness among clinicians and the public, we anticipate significant improvements in surveillance capabilities and more accurate predictions in the future.

## Data Availability

The data analyzed in this study is subject to the following licenses/restrictions: data are available from the authors upon reasonable request and with permission of Provincial Center for Disease Control and Prevention. Requests to access these datasets should be directed to Zheyuan Ding, zhyding@cdc.zj.cn.
