# Supplementary material for: Spatial analysis and prediction of psittacosis in Zhejiang Province, China, 2019–2024
Source: Front Public Health. 2025 Jul 2;13:1604018. doi: 10.3389/fpubh.2025.1604018 (PMC12263594; doi:10.3389/fpubh.2025.1604018)
Supplement: Supplementary file 1 [file Supplementary_file_1.docx]

Supplemental material 1. Mathematical formulation of *Global* and *Local Moran's I*

*Global Moran’s I* is defined as:

$$I=\frac{n\sum_{i=1}^{n} \sum_{j=1}^{n} W_{\mathrm{ij}}(x_{i}-\bar{x})(x_{j}-\bar{x})}{(\sum_{i=1}^{n} \sum_{j=1}^{n} W_{\mathrm{ij}})\sum_{i=1}^{n} {(x_{i}-\bar{x})}^{2}},$$

*Local Moran’s I* is defined as:

$I_{i}=\frac{n(x_{i}-\bar{x})\sum_{j=i}^{n} W_{\mathrm{ij}}(x_{j}-\bar{x})}{\sum_{i=1}^{n} {(x_{i}-\bar{x})}^{2}}$,

where *n* is the number of spatial regions; *x_i_* and *x_j_* is the value of the variable of interest (the incidence of psittacosis cases) on the point *i* and point *j*, respectively; $\bar{x}$ is the mean value of *x*; *w_ij_* is a spatial weight matrix, using inverse distance weighted method.

Supplemental material 2. Mathematical formulation of the semivariogram model and model selection metrics used for the Kriging interpolation

Semivariogram was utilized to estimate attribute values at unmeasured points by measured points separated with a distance *h*. It is defined as:

$$r\left( h \right)=\frac{1}{2N(h)}\sum_{i=1}^{N(h)} {(Z\left( x_{i}+h \right)-Z(x_{i}))}^{2}$$

where *r(h)* is the estimated semivariance for all pairs with a distance h; *N(h)* is the number of pairs with a distance *h*; *Z(x_i_)* is the measured value on point *i*; *Z(x_i_+h)* is the measured value on points with a distance *h* from point *i*.

To determine the most suitable semivariogram model, a semivariogram measurement was computed. Four types of semivariogram functions were considered: Circular, Spherical, Exponential, and Gaussian. Additionally, trend removal using first-, second-, and third-order polynomials was applied within each semivariogram function during the prediction of spatial distribution. This resulted in 16 candidate models for predicting the spatial distribution of cases and four candidate models for predicting cluster risk. The optimal models were selected using the TOPSIS method based on prediction error criteria, which included: 1) the Mean being close to zero; 2) the Mean Standardized being close to zero; 3) the Root-Mean-Square Standardized being close to 1; 4) the Root-Mean-Square being small; 5) the Average Standard Error being small. Ultimately, the Exponential model without trend removal was chosen for predicting incidence, and the Spherical model without trend removal was selected for predicting cluster risk.
